# i-Scan detection of minimal change esophagitis in dyspeptic patients with or without Gastroesophageal Reflux disease

**DOI:** 10.1186/s12876-016-0417-4

**Published:** 2016-01-14

**Authors:** Nisa Netinatsunton, Jaksin Sottisuporn, Siriboon Attasaranya, Teepawit Witeerungrot, Naichaya Chamroonkul, Theeratus Jongboonyanuparp, Alan Geater, Bancha Ovartlarnporn

**Affiliations:** NKC institute of Gastroenterology and Hepatology, Faculty of Medicine, Prince of Songkla University, Hatyai, Songkhla 90110 Thailand; Division of Gastroenterology, Faculty of Medicine, Prince of Songkla University, Hatyai, Songkhla 90110 Thailand; Bangpakok 9 International Hospital, Bangmot, Chom Thonh, Bangkok, 10150 Thailand; Division of Epidermiology, Faculty of Medicine, Prince of Songkla University, Hatyai, Songkhla 90110 Thailand

**Keywords:** Non erosive gastro esophageal reflux disease, Reflux esophagitis, Esophagus

## Abstract

**Background:**

The association of minimal change esophagitis (MCE) with GERD is controversial. i-Scan endoscopy (SE) provides high resolution and modulation of images that may improve minimal change lesion (MCL) detection. We aimed to assess the efficacy of SE in detecting MCL in dyspeptic patients with GERD compared with patients without GERD by GerdQ or by endoscopy with 24-h pH monitoring (PHM) and in normal volunteers.

**Methods:**

This is a cohort study conducted at a tertiary center. All dyspeptic patients were prospectively recruited. All patients completed a validated Thai version of GerdQ and then underwent endoscopy. Forty normal volunteers as a control group were recruited for endoscopy. The distal esophagus was examined by high definition endoscopy and SE sequentially. All had PHM done. GERD was diagnosed by Los Angeles classification A-D and/or by a positive PHM. MCE was diagnosed when MCL or combination of MCL was present.

**Results:**

Of 174 patients, 144 completed the study protocol. After the exclusion of 6 patients, 138 remained for analysis. Overlapping GERD symptoms were found in 44.2 % and 26.8 % had confirmed GERD. Group A was comprised of 61 patients with a positive GerdQ and 77 patients in group B had a negative GerdQ. Twenty-four in group A, 28 in group B and 7 in the control group had MCE that was not significantly different. MCE in GERD was significantly higher (51.45 %) than in non-GERD (32.7 %) (*p* = 0.047) and in the control group (20.58 %) (*p* = 0.007). The sensitivity, specificity, positive predictive value, and negative predictive value of SE were 51.35 %, 67.33 %, 36.54 % and 79.06 %, respectively.

**Conclusion:**

In dyspeptic patients, SE detected more MCE in GERD than in non-GERD patients and in the control group.

**Trial registration:**

ClinicalTrials.gov number NCT01742377

## Background

Gastroesophageal reflux disease (GERD) is a common problem in western communities with a prevalence of 10–20 %. The prevalence of erosive esophagitis (EE) detected by endoscopy varies from 14.8 % to 38 % [1–5]. The prevalence of GERD in Asia including Thailand is lower than in the western countries [4–6]. Overlapping of GERD symptoms in patients with dyspepsia was common with a reported prevalence that varied from 9 % to 42 % [7–9]. The GerdQ questionnaire (GerdQ) is a validated tool for GERD diagnosis and the accuracy of the GerdQ is similar to that of a gastroenterologist, but is better than a primary care physician in GERD diagnosis [10].

Conventional endoscopy had a sensitivity of approximately 40 % for GERD diagnosis [11–13]. The development of new image enhanced technologies such as Narrow Band Imaging, Fujinon Intelligence Color Enhancement (FICE) and i-Scan endoscopy (SE) can provide higher resolution images with image modulation to improve the details of gastrointestinal epithelium and vascular structure which may increase the detection of esophageal minimal change lesions (MCL) that can be used to diagnose minimal change esophagitis (MCE) in GERD [14–19]. Multiple studies of the association of MCE with non-erosive GERD by various endoscopic imaging technologies were reported recently in the literature with conflicting results [14–19].

SE consists of three modes of image enhancement, namely surface enhancement that enhances the mucosal structure, contrast enhancement that digitally adds blue color in relatively dark areas and tone enhancement (TE) that modulates the individual RGB components to create a single new color image. Hoffman, et al. compared the efficacy of high definition (HD) endoscopy, HD endoscopy with SE and HD endoscopy with Lugol’s solution in patients with GERD symptoms and showed that SE helped to identify subtle abnormalities more than HD endoscopy [15]. One study conducted in Thailand in a small number of patients showed that SE detected more MCE in patients with GERD symptoms than in a control group [20]. Another study by Rey JW et al. showed SE detected more MCE in patients with GERD symptoms than in controls with a sensitivity of 82.5 % to detect GERD confirmed by histology [21].

The role of SE in the detection of MCE in dyspeptic patients with or without GERD based on symptoms by the GerdQ or by endoscopy and 24-h pH monitoring (PHM) in a population with a low prevalence of GERD is not well defined. The objectives of this study were to assess the efficacy of SE endoscopy in the detection of MCE in dyspeptic patients with or without GERD diagnosed by GerdQ or by endoscopy plus PHM and in a normal group of volunteers.

## Methods

### Patients

All patients aged more than 18 years with dyspepsia by Rome II criteria [22] with or without symptoms that suggested GERD who were scheduled for endoscopy at the NKC Institute were prospectively recruited from February 2010 to July 2014. Patients with any of the exclusion criteria in Table 1 were excluded. Forty paid healthy volunteers were recruited as a control group for endoscopic examination and PHM study.

**Table 1:** Exclusion criteria in this study

Significant weight loss
Gastrointestinal bleeding
Hematemesis
Melena
Dysphagia
Intractable vomiting
Palpable abdominal mass
History of documented
Peptic ulcer
Gastric cancer
Gastric surgery
Symptoms compatible with irritable bowel syndrome
Hepatobiliary tract disease
Severe concomitant medical conditions
Pregnant woman
Continuous usage of NSAID^a^ in the preceding 1 month before entry to the study

^a^Non-steroid anti-inflammatory drugs

This study was conducted at NKC institute of Gastroenterology and Hepatology. This study was conducted according to the Declaration of Helsinki and approved by the ethics committee at the Faculty of Medicine, Prince of Songkla University.

### Study protocol

A Thai version of GerdQ was translated from the English version [10]. Contents of the Thai GerdQ were tested by back translation from Thai to English by 5 personnel who were fluent in English and all showed consistent content. The reliability of the GerdQ was validated in 22 volunteers completing the questionnaire twice with 3 hours in between and showed no significant difference in the mean scores (5.14 ± 2.34 versus 6.23 ± 1.57, *p* = 0.07) and the number of subjects with GERD diagnosed by GerdQ (2 versus 5, Fisher’s exact test, *p* = 0.21). All patients signed an informed consent prior to participation in the study and all patients completed the Thai version of GerdQ provided by endoscopic nursing staff. A GerdQ score equal to or more than 8 is a positive score for GERD [10]. All of the healthy volunteers provided a signed consent but did not take part in the questionnaire session.

**Fig. 1:**
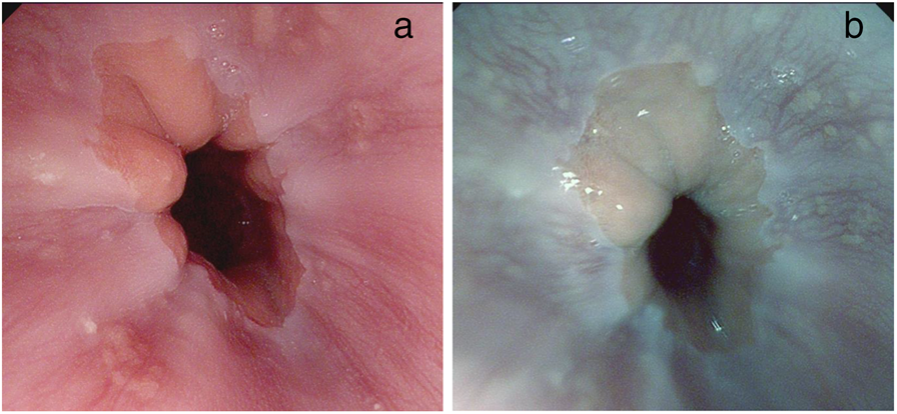
High definition image (**a**) and SE TE-e mode image (**b**)

**Fig. 2:**
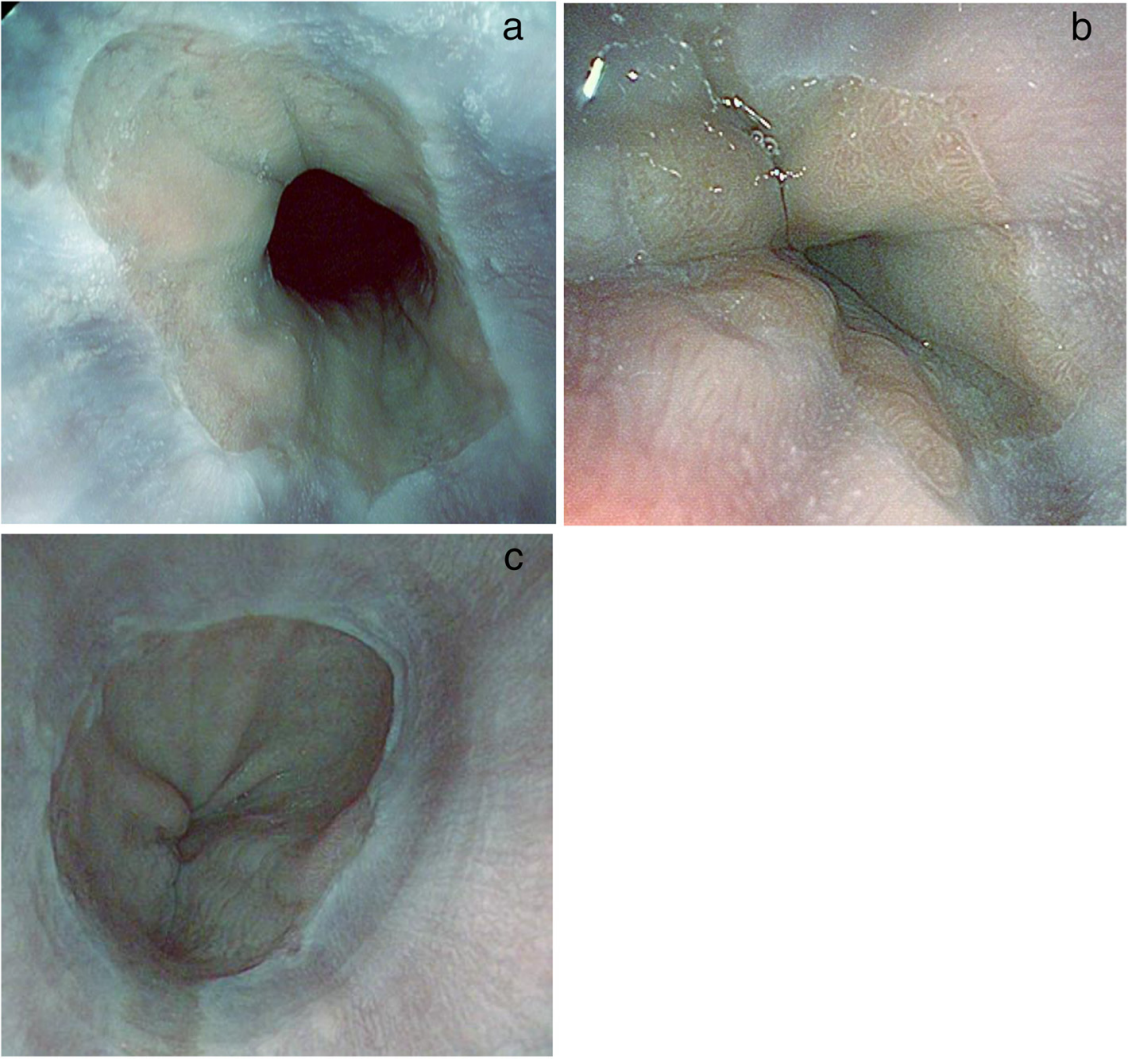
A punctate erythema (**a**), gastric mucosa with elongated pit pattern and triangular lesion at squamocolumnar junction (**b**), and a short gastric mucosa tongue with minute erosion (**c**) by SE endoscopy TE-e mode

**Fig. 3:**
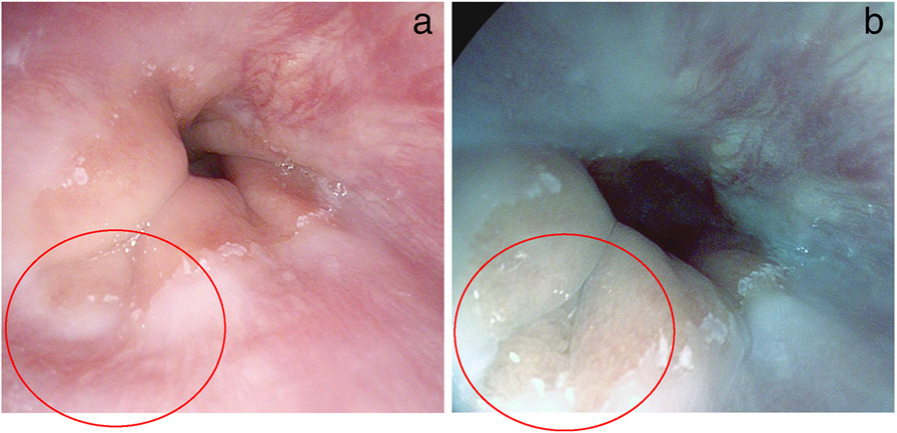
Blurred palisade vessels at squamocolumnar junction by high definition endoscopy (**a**) and by SE endoscopy (**b**)

Endoscopic examination was done using a Pentax Model EG-2990i Gastroscope with EPKi processor under conscious sedation with intravenous midazolam and meperidine. All of the endoscopists were blinded to the GerdQ score results. Fifteen minutes before the endoscopic examination, the patient swallowed a suspension of simethicone. Additional flushing during the procedure was done as required to get rid of bubbles and/or mucus. The distal esophagus was inspected by HD endoscopy and followed by SE with a preset TE mode for esophagus (TE-e mode) (Fig. 1). All images were captured in a high resolution mode and stored on a flash drive for later inspection by two additional endoscopists who were blinded to the results of the first endoscopist’s report. The endoscopic diagnosis of esophagitis and grading were based on the Los Angeles (LA) classification [23]. The MCL detected by SE in TE-e mode included punctate erythema (PE), elongated pit pattern of gastric mucosa with triangular lesion (EP) at the squamocolumnar junction, minute erosion (ME) and blurred palisade blood vessel at the distal esophagus adjacent to the Z-line [24, 25] (Fig. 2 and Fig. 3). The agreement of at least two endoscopists was accepted as the final result. The location of the Z-line was recorded during the endoscopy while withdrawing the endoscope.

All patients and volunteers had a PHM study within 1 week after the endoscopy. The PHM was done with a Digitrapper^R^ pH400 (Medtronic, Denmark) or AccuTrac ^pH-Z^ (Sierra Scientific Instruments) with a pH probe placed at 6 cm above the Z-line [26, 27]. Any use of proton pump inhibitor was stopped at least 1 week before the study. Data of the 24-h pH monitoring were analyzed by commercial computer software (Medtronic, Sierra Scientific Instruments).

The PHM data of the 40 volunteers were analyzed to set the upper normal limit of total acid exposure time in our population. Six volunteers were excluded. Two had erosive esophagitis and four had poor quality images which left 34 volunteers for the PHM data and endoscopic findings analysis. The mean + 2SD of total acid exposure time of the volunteers was 1.9 % (data shown below). The upper limit of total acid exposure was set at 2 % in our population.

GERD was diagnosed when total acid exposure time was more than 2 % and/or the symptom index was positive and/or the symptom association probability was positive and/or the endoscopy revealed LA erosive esophagitis ≥ grade A [23, 26, 27].

Our interim analysis in 89 patients that compared HD endoscopy and SE showed that SE was significantly superior to HD endoscopy in detecting MCL [28]. Therefore, we finally used the SE images for analysis in this study.

The endoscopic images by SE with the TE-e mode in 34 volunteers showed blurred palisade blood vessels in 32 of the 34 volunteers, so this lesion was not included in our analysis of MCL. MCE was diagnosed based on the presence of elongated pit pattern of gastric mucosa at the squamocolumnar junction or ME or PE or any combination of these lesions detected by SE with the TE-e mode. The analysis of inter- and intra-observer variation for the interpretation of endoscopic lesions by SE at our institute was reported elsewhere [29].

### Data sharing

The individual data of patients participating in this study are not available for public sharing since we did not obtain the consent to share the data of the patients.

### Statistical Analysis

Data were expressed as mean with standard deviation and as frequency with percentage where appropriate. The chi-square test was used for comparisons between two groups. A *p*-value equal to or less than 0.05 was considered as statistically significant. The statistical analysis used Minitab® 15 software.

## Results

**Fig. 4:**
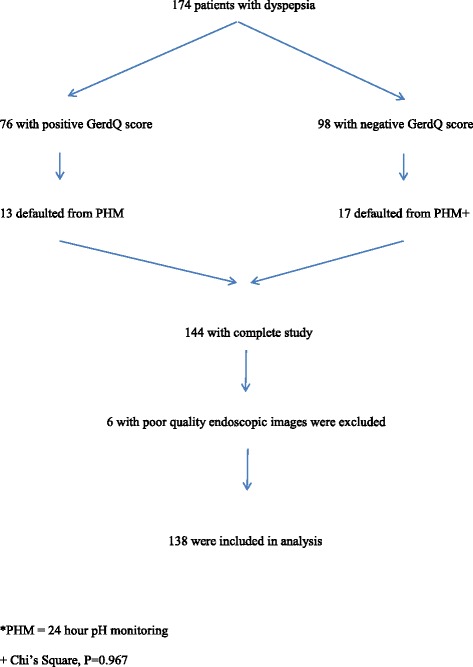
The flow diagram of patients included in the analysis

One hundred and seventy-four patients (52 males and 122 females) were recruited in this study. The mean age ± SD of the patients was 46.81 ± 12.36 years with a range of 20–75 years. The mean ± SD of duration of dyspeptic symptoms was 28.21 ± 33.82 months (range 2–120 months). One hundred and forty-four patients completed the study protocol and 30 patients did not have the pH study done. Seventy-six patients had a positive GerdQ score with 13 patients who defaulted from the PHM. Ninety-eight patients had a negative GerdQ score with 17 patients who defaulted from the PHM. The number of defaulted patients between the two groups was not significantly different (Chi-square, *p* = 0.967). Six patients in the completed study group were excluded due to poor quality endoscopic images (Fig. 4).

One hundred and thirty-eight patients were included in the final analysis; 95 were females and 43 were males with a mean age ± SD of 46.88 ± 12.84 years (range 20–75 years). Overlapping GERD symptoms were present in 61 (44.2 %) of 138 patients and the number with confirmed GERD for the whole group was 37 (26.8 %) of 138. The endoscopic findings are shown in Table 2 and the majority of patients had functional dyspepsia.

**Table 2:** The endoscopic findings of 138 patients

Normal	103
Erosive gastritis/non-specific gastritis/hemorrhagic gastritis	25
Exudative duodenitis	3
Gastric polyp	5
DU	1
Erosive esophagitis	1

Sixty-one in group A had a positive GerdQ score, and 77 in group B had a negative GerdQ score. The prevalences of PE, ME, and EP in group A, group B, and normal control subjects in patients with GERD and patients without GERD are shown in Tables 3 and 4. The prevalence of each individual lesion was not statistically different among the groups. MCE was detected in 24 cases in group A and in 28 cases in group B, but the difference was not significant (chi-square, *p* = 0.720). The number of patients with GERD in group A was 20 (32.8 %) and it was higher than 17 (22 %) in group B, but the difference was not statistically significant (Chi’s square *p* = 0.158) (Table 3). The 51.4 % prevalence of MCE (19 of 37) in patients with GERD was significantly higher than the 32.7 % prevalence of MCE (33 of 101) in patients without GERD (chi-square, *p* = 0.047). The sensitivity, specificity, PPV and NPV for MCE in detecting GERD were 51.35 %, 67.33 %, 36.54 % and 79.06 %, respectively.

**Table 3:** Prevalence of MCL, MCE, and GERD in group A, B and volunteer

	Group A (*n* = 61)	Group B (*n* = 77)	Volunteer (*n* = 77)	*P*-value
Endoscopic findings (n)				
MCE	24	28	7	0.160
PE	14	18	4	0.340
ME	3	1	3	0.143
EP	11	16	6	0.891
Confirmed GERD	20	17		0.158

*P*-values from chi square or Fisher’s exact test, as appropriate

**Table 4:** Prevalence of MCL, and MCE in patients with and without GERD and in volunteer

	Confirmed GERD (*n* = 37)	Non-GERD (*n* = 101)	Volunteer (*n* = 34)	*P*-value
Endoscopic findings (n)				
MCE	19^a^	33^a^	7^b^	0.021
PE	10	22	4	0.271
ME	2	2	3	0.195
EP	10	17	6	0.391

*P*-value from chi square or Fisher’s exact test, as appropriate

^ab^ Proportions not having a superscript in common differ significantly (*p* < 0.05)

### MCE and total acid exposure in patients compared with normal volunteers

Seven of 34 healthy volunteers had MCE by endoscopy. This was not significantly different from 24 in 61 in group A or 28 in 77 in group B (*p* = 0.056, 0.091) (Table 3). The mean ± SD of total acid exposure time in 34 volunteers was 0.474 ± 0.717 %. The mean ± SD of total acid exposure time in patients with positive GerdQ was 1.62 ± 2.66 %. This was significantly higher than the volunteers (*p* = 0.002). The mean ± SD of total acid exposure time in patients with negative GerdQ was 1.00 ± 2.2 %, but this was not statistically significant from that of the volunteers (*p* = 0.063). The total acid exposure time in group A was not significantly different from that of group B (*p* = 0.148). The prevalence of MCE in 19 of 37 patients with GERD was significantly higher than in the volunteers (7 of 34), but the prevalence of MCE in 33 of 101 non-GERD patients was not significantly different from that of the volunteers (Table 4).

## Discussion

The prevalence of 44.2 % of overlapping GERD symptoms in our dyspeptic patients was within the 9–42 % range reported in other studies [7–9]. The prevalence of 26.8 % of confirmed GERD for all patients in this study was similar to 23 % reported in the study of Tack et al. [7]. However, the prevalence of 32.8 % of confirmed GERD in patients with GERD symptoms in our study was much lower than the prevalence of 76 % confirmed GERD in patients with heartburn in the study of Tack et al. [7]. Nevertheless, the prevalence of 22 % of confirmed GERD in patients without GERD symptoms in this study was similar to the prevalence of 18.5 % in the study of Tack et al. [7]. The majority of the patients with overlapping GERD symptoms in this report had functional heartburn based on conventional PHM and endoscopic examination. The prevalence of 0.72 % EE in this study was much lower than the 14.8 % to 38 % reported in other studies [3–5]. The prevalence of GERD in the population in question may affect the outcome of GERD diagnosis. The population in our study is in a region where the prevalence of GERD is low [6] with concomitant symptoms of dyspepsia so these symptoms may increase the probability of recruiting more patients with functional heartburn. However, the mean total acid exposure time in patients with positive GerdQ score was significantly higher than in controls. The higher acid exposure time, even though it was not in the range to diagnose GERD by conventional PHM criteria, may be a factor to explain the symptoms. Moreover, esophageal hypersensitivity to a small amount of acid may also contribute to the development of symptoms.

The results of studies regarding the association of MCE with non-erosive GERD reported in the literature are conflicting [24, 25, 30–34]. The individual lesion of MCL had no discriminating power in any group of patients in our study. The proportions of patients with MCE detected by SE with the TE-e mode in patients with positive GerdQ in group A, in patients with negative GerdQ in group B and in the volunteers were not significantly different in this report. Nevertheless, one study by Pisespongsa et al. reported a significantly higher number of MCE by a preset TE mode for colon (TE-c) in 27 patients with GERD by symptom-based diagnosis than in 21 controls in a Thai population [20]. Another study by Rey JW et al. [21] showed that SE detected MCE significantly more often in patients with GERD symptoms than in the control group. A different mode of SE used or a different type of MCL or a different population may account for the discrepancies between the present study and the other studies of SE [20, 21]. In another study from Thailand that used FICE in 21 patients with GERD symptoms compared with 9 control subjects, showed a higher prevalence of MCE than in the control group [19]. In a study by Kim et al. in 1445 patients, the prevalence of MCE was not significantly different between patients with GERD and patients without GERD by the GerdQ score, but another study from Korea showed that MCE may be associated with GERD by a symptom-based diagnosis [30, 31]. The prevalence of MCE in our study was significantly higher in patients with GERD than in patients without GERD and the volunteers. A study by Edebo et al. [25] in patients with GERD confirmed by PHM showed that MCE was associated with GERD but the sensitivity and specificity of MCE were not sufficient to justify MCE as a diagnostic criterion for non-erosive GERD. Our study also showed a low sensitivity and specificity of MCE in the diagnosis of GERD supporting the finding of Edebo et al. [25]. However, Rey et al. [21] reported a sensitivity of 82.5 % and a positive predictive value of 98.3 % for SE in detecting MCE in 65 patients with reflux symptoms. The discrepancy may be due to a different method for GERD diagnosis since Rey et al. used histological examination as a gold standard whereas our study used 24–hour pH monitoring for GERD diagnosis. Moreover, our population had concomitant dyspeptic symptoms which may not represent a population with only GERD symptoms. The lack of a standardized definition for MCE, no definite gold standard tool for GERD diagnosis, different populations recruited, different endoscopic image technologies and the low inter-observer agreement for endoscopic minimal changes may account for different results from the reports in the literature [25, 29, 31].

The clinical implications of detecting MCE for patient management in terms of disease behavior and treatment response as well as cost benefits have not been evaluated before and needs further study.

This study had some limitation. It was a single tertiary center study. The population in this study had a selection bias toward the recruitment of more functional heartburn patients. No histologic evaluation of the MCL was done and an impedance study that may detect weak acid or non-acid reflux was done in only a few patients.

## Conclusion

The prevalence of MCE detected by SE with the TE-e mode was similar in dyspeptic patients with or without overlapping GERD symptoms and in the volunteers. However, SE had a higher detection rate for MCE in dyspeptic patients with GERD by endoscopy and/or PHM than in non-GERD patients and in the normal control group. Nevertheless, the sensitivity and specificity of MCE detected by SE were low, so it is not suitable as a clinical tool to diagnose GERD in our population.

## Abbreviations

EE: Erosive Esophagitis
EP: Elongated Pit Pattern of gastric mucosa with triangular lesion
FICE: Fujinon Intelligence Color Enhancement
GERD: Gastro Esophageal Reflux Disease
GerdQ: GerdQ Questionnaire
HD: High Definition
LA: Los Angeles classification
MCL: Minimal Change Lesions
MCE: Minimal Change Esophagitis
ME: Minute Erosion
PE: Punctate Erythema
PHM: 24-h pH monitoring
SE: i-Scan Endoscopy
TE: Tone Enhancement
TE-e: Tone Enhancement Mode for Esophagus
TE-c: Tone Enhancement Mode for Colon
